# Loss of LECT2 promotes ovarian cancer progression by inducing cancer invasiveness and facilitating an immunosuppressive environment

**DOI:** 10.1038/s41388-023-02918-w

**Published:** 2024-01-04

**Authors:** Chin-Jui Wu, Ke-Fan Pan, Ji-Qing Chen, Yu -Chen Tao, Yu-Cheng Liu, Bo-Rong Chen, Ching Hsu, Ming-Yang Wang, Bor-Ching Sheu, Michael Hsiao, Kuo-Tai Hua, Lin-Hung Wei

**Affiliations:** 1 Department of Obstetrics and Gynecology, National Taiwan University Hospital, College of Medicine, National Taiwan University, Taipei, Taiwan; 2 Department of Obstetrics and Gynecology, National Taiwan University Hospital Hsin-Chu Branch, Hsin-Chu City, Taiwan; 3 Graduate Institute of Toxicology, College of Medicine, National Taiwan University, Taipei, Taiwan; 4 Department of Medical Education and Research, Wan Fang Hospital, Taipei Medical University, Taipei, Taiwan; 5 Division of General Surgery, Department of Surgery, Wan Fang Hospital, Taipei Medical University, Taipei, Taiwan; 6 Division of Colorectal Surgery, Department of Surgery, Wan Fang Hospital, Taipei Medical University, Taipei, Taiwan; 7 Department of Epidemiology, Geisel School of Medicine at Dartmouth, Lebanon, NH 03756 USA; 8 Department of Surgery, National Taiwan University Hospital, Taipei, 100 Taiwan; 9 Genomics Research Center, Academia Sinica, Taipei, Taiwan; 10 Department of Medical Research, China Medical University Hospital, China Medical University, Taichung, Taiwan

**Keywords:** Ovarian cancer, Prognostic markers, Cancer microenvironment

## Abstract

Leukocyte cell-derived chemotaxin 2 (LECT2) is a multifunctional cytokine that can bind to several receptors and mediate distinct molecular pathways in various cell settings. Changing levels of LECT2 have been implicated in multiple human disease states, including cancers. Here, we have demonstrated reduced serum levels of LECT2 in patients with epithelial ovarian cancer (EOC) and down-regulated circulating Lect2 as the disease progresses in a syngeneic mouse ID8 EOC model. Using the murine EOC model, we discovered that loss of Lect2 promotes EOC progression by modulating both tumor cells and the tumor microenvironment. Lect2 inhibited EOC cells’ invasive phenotype and suppressed EOC’s transcoelomic metastasis by targeting c-Met signaling. In addition, Lect2 downregulation induced the accumulation and activation of myeloid-derived suppressor cells (MDSCs). This fostered an immunosuppressive microenvironment in EOC by inhibiting T-cell activation and skewing macrophages toward an M2 phenotype. The therapeutic efficacy of programmed cell death-1 (PD-1)/PD-L1 pathway blockade for the ID8 model was significantly hindered. Overall, our data highlight multiple functions of Lect2 during EOC progression and reveal a rationale for synergistic immunotherapeutic strategies by targeting Lect2.

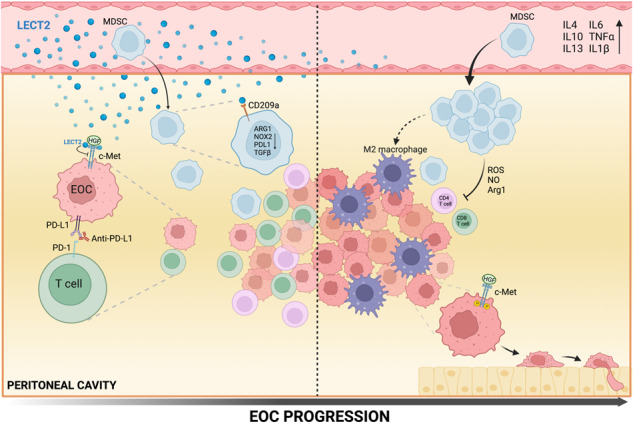

## Introduction

Epithelial ovarian cancer (EOC) remains one of the most significant clinical challenges in medicine. Most EOC cases are discovered when the primary tumor is disseminated via the transcoelomic route [1]. At staging laparotomy, approximately 70% of patients have multifocal intraperitoneal metastasis and malignant ascites. About 75% of patients with advanced EOC develop recurrent disease, which is generally not curable [2]. New treatment strategies and paradigms are still urgently needed for EOC.

Immune checkpoint blockade (ICB) antibodies can unleash anti-tumor immunity and mediate durable cancer regressions in patients with various malignancies. However, the results of multiple studies of ICB therapy in advanced EOC have been disappointing, and EOC remains cancer with no ICB-specific approvals. Immune rejection of EOC has been demonstrated in several pre-clinical animal models; correlative human studies in patients with EOC strongly support the role of immune system involvement in patient outcomes [3]. An evolving understanding of the mechanisms driving low response rates to ICB in EOC, including tumor cell-intrinsic features such as low total tumor burden (TMB) and lack of an inflammatory gene expression profile [4], suppressed major histocompatibility complex protein expression [5], as well as a generalized immunosuppressive tumor immune microenvironment [6]. Studies addressing these issues are much needed to provide insights into developing novel therapeutic strategies for EOC.

Human leukocyte cell-derived chemotaxin 2 (LECT2) is a 16 kDa secreted protein initially identified as a chemotaxin of neutrophils [7]. LECT2 protein is found mainly in the cytoplasm of human hepatocytes before secretion [8]. Lect2-deficient mice livers have increased invariant natural killer T cells(iNKT) and excessive IL-4 and Fas ligand expression, suggesting an anti-inflammatory action of Lect2 [9]. The human *LECT2* gene has been mapped to chromosome 5q31.1-q32, a cluster harboring several genes encoding immunomodulatory cytokines. Previous studies demonstrated that LECT2 improves protective immunity in bacterial sepsis, possibly due to enhanced macrophage functions via the CD209a receptor [10]. Clinical and animal model evidence supports a tumor-suppressive role of LECT2 in cancer, particularly hepatocellular carcinoma [11, 12]. However, the pathologic role of LECT2 in other cancer types is unclear and left largely unexplored.

Substantial data support the presence of immune cell infiltration in EOC and its microenvironment. Large numbers of monocytes/macrophages are present in the ascitic fluid, which may comprise 50% or more of the mononuclear leukocyte population. In contrast, the proportion of T-lymphocytes is usually below 40% [13]. Evidence suggests that infiltrating immune cells may enhance immunity or tumor growth and progression. Furthermore, we and our colleagues have found that LECT2 directly binds to the α chain of the MET extracellular domain and inhibits MET signaling by recruiting PTP1B [12]. The activation of hepatocyte growth factor (HGF)/mesenchymal-epithelial transition factor (c-MET) signaling promoted peritoneal dissemination and invasion of EOC and was associated with a poor prognosis [14, 15]. Through this observation, we explore the potential role of LECT2 in EOC and map out its action in EOC progression.

## Results

### Reduction of circulating LECT2 was significantly associated with EOC progression in humans and a syngeneic mouse model

**Fig. 1:**
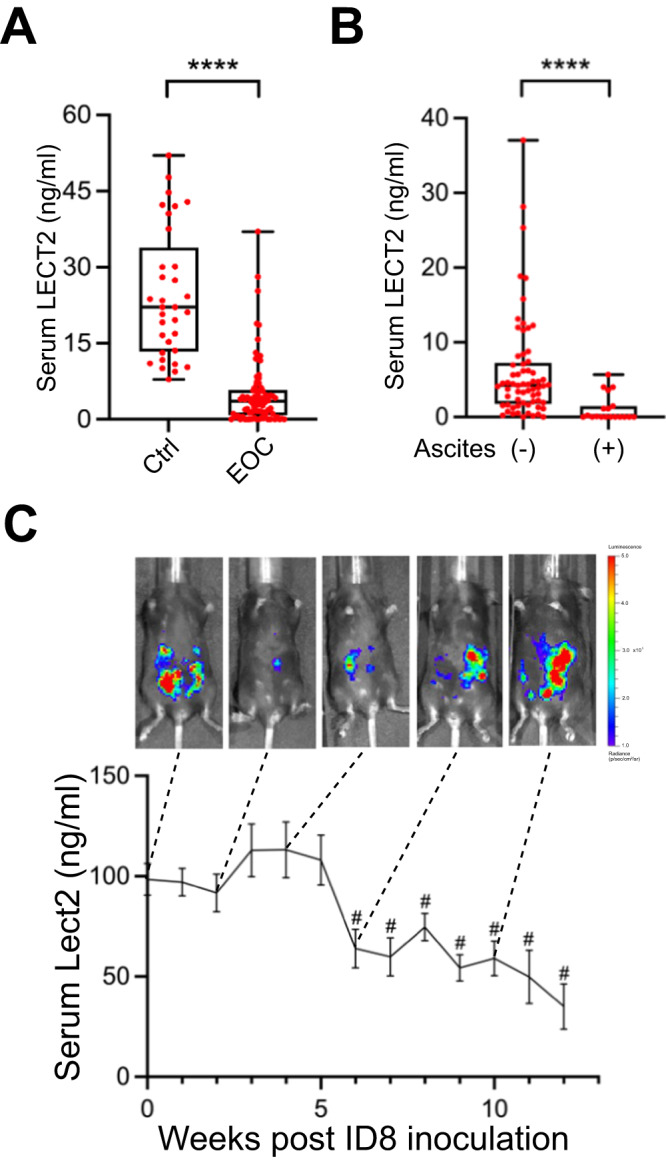
In vivo LECT2 concentration of EOC. **A** Comparison of serum levels of LECT2 protein in EOC (*n* = 84) and healthy women (*n* = 33). ****P < 0.0001. **B** The LECT2 level in EOC patients with malignant ascites (*n* = 19) and without detectable ascites (*n* = 65). ****P < 0.0001. **C** Serial detection of Lect2 in the intraperitoneal ID8/luciferase mice model. A drop in serum Lect2 level was measured after week 6, as shown in representative pictures of increased peritoneal luminescence. ^#^*P* < 0.0001.

We examined the amount of circulating LECT2 protein in the serum collected from 84 EOC patients and 33 healthy volunteers. Clinical and pathological characteristics of patients with EOC are shown in Supplementary Table 2. The level of serum LECT2 protein was significantly lower in the EOC patients than in the healthy women (*P* < 0.0001; Fig. 1A). Additionally, when we grouped EOC patients according to whether they had malignant ascites, an even tinier amount of serum LECT2 was detected in those patients with malignant ascites (*P* < 0.0001; Fig. 1B). Next, we monitored the serum concentration of Lect2 in an ID8 syngeneic mouse EOC model. The serum Lect2 level remained stable in the 98.5 ± 8 ~ 108 ± 12.4 ng/ml during the first five weeks after intraperitoneal inoculation of ID8/Luc cells. In this period, the in vivo luciferase images show relatively weak and focused signals compared with solid and dispersed signals taken after injection. Notably, we discovered that the serum Lect2 concentration started to drop significantly beginning in the 6th week (*P* < 0.0001) after injection, accompanied by increased luciferase signals in the peritoneal cavity (Fig. 1C).

### Lect2 deletion promoted transcoelomic metastasis and malignant ascites formation in mice with EOC

**Fig. 2:**
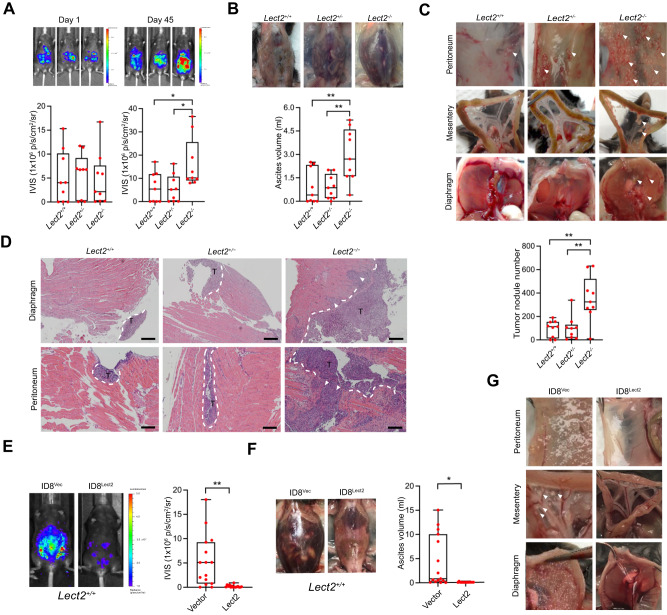
*Lect2*^−/−^ mice are susceptible to transcoelomic metastasis of EOC. **A** The ID8/Luc cells in mice at day 0 and day 45 after inoculation. The luminescence intensity represents the tumor burden of the *Lect2*^−/−^, *Lect2*^+/+^, and *Lect2*^+/−^ groups. *P < 0.05. **B** The ascites volume of the mice. Representative pictures of ascites taken on day 45 after the sacrifice are shown above the plot. **P < 0.01. **C** Quantitative plot and representative pictures of the metastatic tumors (white arrowheads) distributed in the abdominal cavity, including those on the peritoneum, mesentery, and diaphragm, among mice. **P < 0.01. **D** Representative H&E staining pictures of the tumor-containing areas in the diaphragm and peritoneum from mice. White dotted line: tumor/tissue interface. White arrowheads: the tumor invasive front. T: tumor, scale bar: 500 μm. **E** The tumor burden of *Lect2*^+/+^ mice intraperitoneally inoculated with ID8/*Lect2* or the controlled ID8/Vector cells for 10 weeks. Representative luminescence photos and quantitative plots are shown. **P < 0.01. **F** The ascites volume of the *Lect2*^+/+^ mice inoculated with ID8/*Lect2* and the control ID8/Vector cells. *P < 0.05. **G** Representative pictures of tumors (white arrowheads) in the peritoneum, mesentery, and diaphragm of the *Lect2*^+/+^ mice inoculated with ID8/*Lect2* and the controlled ID8/Vector cells.

We evaluated the tumor development of ID8/Luc cells in *Lect2*-deficient C57BL/6J mice (*Lect2*^+/−^ and *Lect2*^−/−^) and in their wild-type littermates (*Lect2*^+/+^). Notably, the tumor burden was significantly higher in the *Lect2*^−/−^ mice after 45 days of inoculation than in the wild-type (*P* < 0.05) or the heterogeneous mice (*P* < 0.05; Fig. 2A). In addition, considerably more ascites, which were highly associated with the advanced disease status of EOC, can be observed in *Lect2*^−/−^ mice than in *Lect2*^+/+^ or *Lect2*^+/−^ mice (*P* < 0.01; Fig. 2B). The metastatic tumors distributed in the abdominal cavity, including those on the peritoneum, mesentery, and diaphragm, were significantly more than those in the *Lect2*^+/−^ or *Lect2*^+/+^ mice (*P* < 0.01; Fig. 2C). Moreover, the ID8 tumors developed in the *Lect2*^−/−^ mice show a prominent invasive front and spread into the surrounding tissues, while tumors in *Lect2*^+/−^ and *Lect2*^+/+^ mice have smaller sizes and a smoother front (Fig. 2D). To further examine whether manipulating Lect2 levels may benefit EOC, we established ID8/Luc cells with *Lect2* stably overexpression. We subjected them to the intraperitoneal injection model of EOC inoculation with *Lect2*^+/+^ mice. Compared to the control group, the ID8/*Lect2* group had a significantly smaller tumor burden (*P* < 0.01), less peritoneal seeding metastases, and more limited ascites formation (*P* < 0.05) (Fig. 2E–G). Our results demonstrated the significant role of Lect2 in suppressing EOC progression.

### Lect2 regulated EOC cell adhesion, migration, and invasion by inhibiting c-Met signaling

**Fig. 3:**
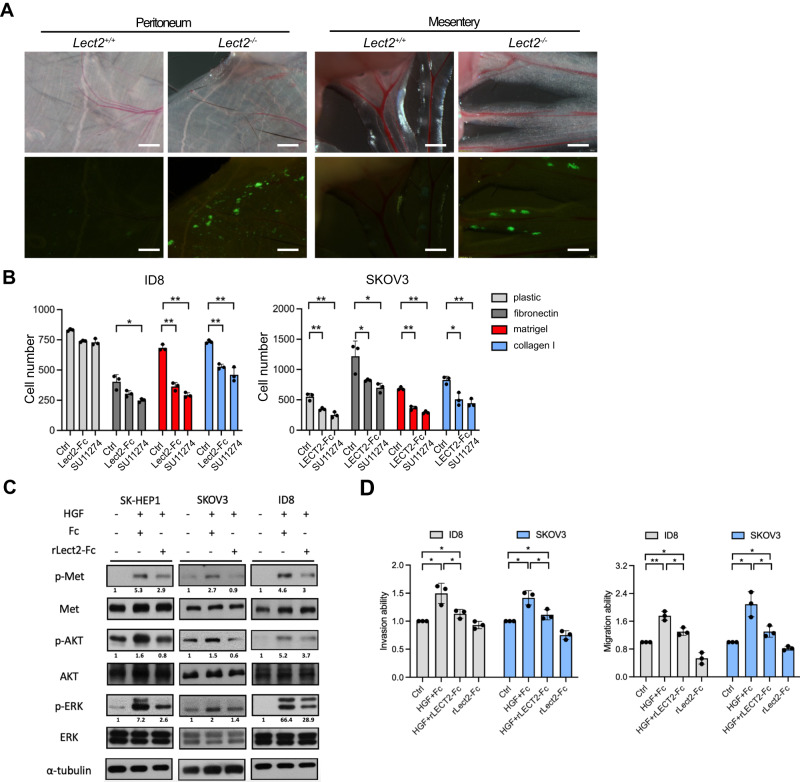
Recombinant Lect2 protein suppresses the HGF/c-Met signaling axis in ovarian cancer cells. **A** The in vivo adhesion abilities of CMFDA-labeled ID8 cells in the peritoneum and mesentery were dissected from mice 24 h after injection. Scale bar, 500 μm. **B** CMFDA-labeled cells were treated with recombinant LECT2/Lect2 proteins (200 ng/ml) or SU11274 (Met inhibitor, 10 nM). After incubation for 1 h at 37°C, attached cells were counted. **C** Western blot analysis indicates that HGF (30 ng/ml) activates the c-Met tyrosine kinase pathway, while recombinant Lect2 (200 ng/ml) mitigates HGF/c-Met signaling. Western blot quantifications using ImageJ were marked below the indicated molecules. **D** The suppression ability of recombinant LECT2/Lect2 proteins in the transwell migration and invasion assays of HGF-induced SKOV-3 and ID8 cells. HGF (30 ng/ml), recombinant LECT2/Lect2 (200 ng/ml), and Fc (200 ng/ml) were used as indicated. In (**C**) & (**D**), human and mouse recombinant LECT2/Lect2 were used for the human and mouse cell lines, respectively. *P < 0.05 and **P < 0.01.

C-Met overexpression has a significant prognostic impact on EOC [15, 16]. We have previously discovered that LECT2 acts as an antagonist of c-Met in hepatocellular carcinoma [12]. Thus, we investigated if Lect2 influences the aggressive phenotype of EOC through effective inhibition of c-Met-mediated signaling. We observed more ID8 cells adhering to the peritoneum and mesentery in the *Lect2*^−/−^ mice at 24 h (Fig. 3A). The recombinant LECT2/Lect2 (rLECT2/rLect2) proteins significantly suppressed the adhesive potential of human and murine EOC cells, the SKOV-3 and ID8 cells, respectively, to different extracellular components (Fig. 3B). Besides, the selective c-Met kinase inhibitor SU11274 suppressed the adhesion abilities of both EOC cell models. rLECT2/rLect2 also diminished the HGF-induced c-Met phosphorylation and the downstream signaling molecules, phosphorylated AKT and ERK, in SKOV-3 and ID8 cells (Fig. 3C). Notably, rLECT2/rLect2 proteins also significantly suppressed the HGF-induced migration, invasion, and adhesion of SKOV-3 and ID8 cells (Fig. 3D and Supplement Fig. 1).

### Targeting the c-Met signaling pathway suppresses EOC metastasis in *Lect2*^−/−^ mice

**Fig. 4:**
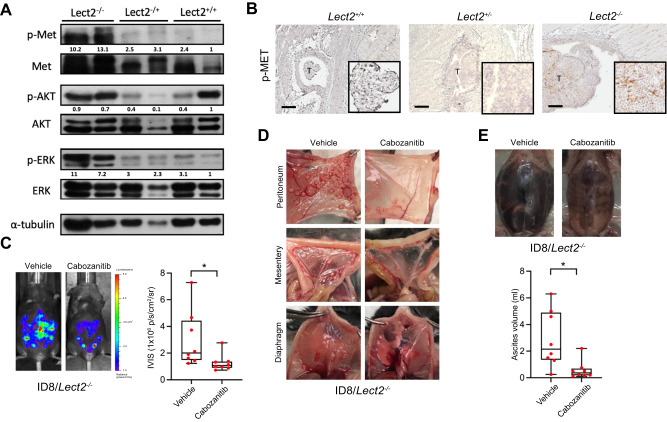
Targeting the c-Met signaling pathway suppresses EOC metastasis potential and tumor invasiveness in the *Lect*2^−/−^ mice. **A** Western blot analysis shows the activation and expression of the c-Met pathway in the tumors dissected from mice. Western blot quantifications using ImageJ were marked below the indicated molecules. Tumors of 2 mice in each group are shown. **B** The immunohistopathologic staining of phosphorylated-Met (p-Met) in mice is brown. T: tumor, scale bar: 500 μm. **C** *Lect2*^*−/−*^ mice were intraperitoneally injected with ID8 cells. After one week, mice were treated with 30 mg/kg Cabozantinib or vehicle 5 days a week for 6 weeks. The representative photos and quantitative plot show the tumor burden in these mice before sacrifice. *P < 0.05. **D** Representative pictures of tumors from the Cabozantinib- and vehicle-treated group present in the peritoneum, mesentery, and diaphragm of Lect2^−/−^ mice. **E** Representative photos and quantitative plots show the ascites volume of *Lect2*^*−/−*^ mice from the Cabozantinib- and vehicle-treated group. *P < 0.05.

Next, we examined the c-Met phosphorylation levels of the peritoneal tumors obtained in mice with different Lect2 genotypes. The Western blot and immunohistochemical staining demonstrated that the phosphorylated c-Met levels were higher in cancer obtained from *Lect2*^−/−^ mice than *Lect2*^+/+^ or *Lect2*^+/-^ mice (Fig. 4A, B). The classical c-Met downstream signaling molecules, including p-Akt and p-Erk, were higher in the tumor specimens removed from *Lect2*^−/−^ mice (Fig. 4A). We showed that the treatment of Cabozantinib, a potent c-Met inhibitor, significantly inhibited the ID8 tumor burden in the peritoneal cavity of *Lect2*^−/−^ mice (*P* < 0.05; Fig. 4C). Cabozantinib also drastically eliminated ID8 tumor seeding of the peritoneum, mesentery, and diaphragm in *Lect2*^−/−^ mice (Fig. 4D). Moreover, significantly lower ascites formation was observed in the Cabozantinib treatment *Lect2*^−/−^ mice compared with the vehicle treatment group (*P* < 0.05; Fig. 4E).

### Loss of Lect2 facilitated tumor-promoting inflammation and immunosuppression during EOC progression

**Fig. 5:**
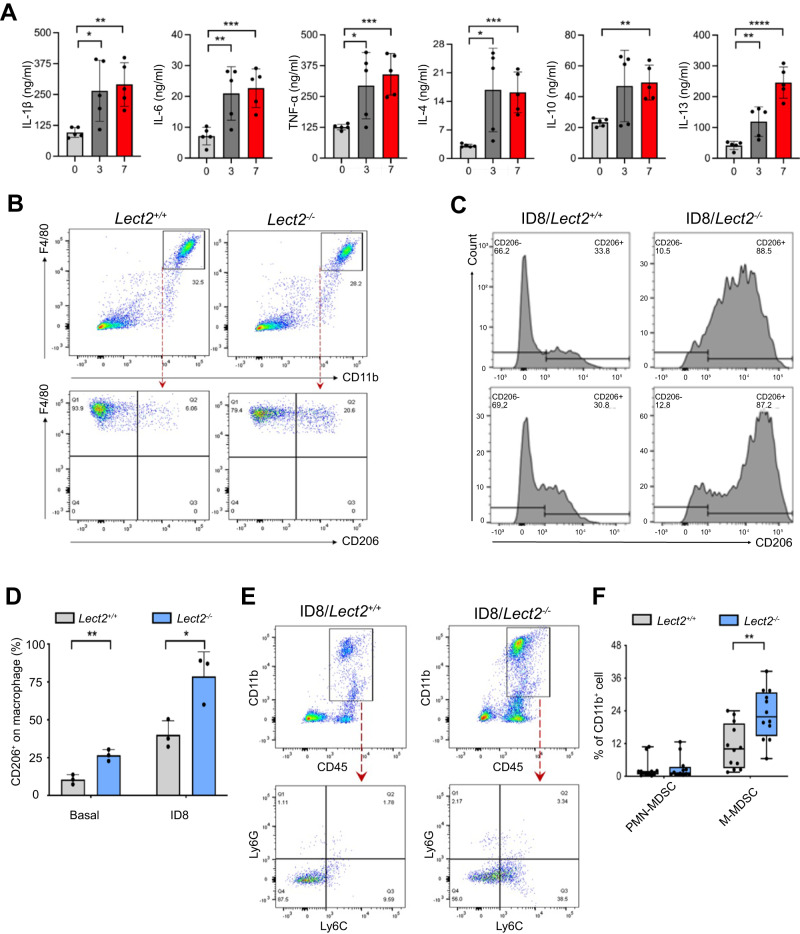
Loss of Lect2 increases tumor-promoting inflammation. **A** The levels of immunosuppressive (IL-4, IL-10, and IL-13) and pro-inflammatory (IL-6, IL-1β, and TNF-α) cytokines detected at weeks 0, 3, and 7 during EOC progression in the same ID8 intraperitoneal inoculation model as shown in Fig. 1C. *P < 0.05, **P < 0.01, ***P < 0.001, and ****P < 0.0001. **B** The representative density plots from flow cytometry analysis of peritoneal cells harvested by peritoneal lavage indicate the proportion of CD11b^+^F4/80^+^CD206^+^ cells in *Lect2*^−/−^ and *Lect2*^+/+^ mice. **C** Accumulation of CD11b^+^F4/80^+^CD206^+^ cells in the malignant ascites of ID8^/^*Lect2*^−/−^ and ID8/*Lect2*^+/+^ mice. Peritoneal cells were harvested 5 weeks after ID8 inoculation and analyzed by flow cytometry. **D** The bar chart of CD206^+^ macrophage proportions with or without ID8 tumor challenge in *Lect2*^−/−^ and *Lect2*^+/+^ mice. *P < 0.05 and **P < 0.01. **E** The representative density plots from flow cytometry analysis gated on the abundance of CD45^+^CD11b^+^Ly6C^+^Ly6G^-^ (M-MDSC) and CD45^+^CD11b^+^Ly6C^-^Ly6G^+^ cells (PMN-MDSC) in ID8^/^*Lect2*^−/−^ and ID8/*Lect2*^+/+^ mice. **F** Quantification of the ascitic M-MDSC and PMN-MDSC in ID8/*Lect2*^**−**/**−**^ and ID8/*Lect2*^+/+^ mice. **P < 0.01.

We further investigated whether Lect2 possesses immunostimulatory properties and suppresses cancer-promoting inflammation of EOC using a syngeneic ID8 mouse model. Along with the loss of Lect2 during EOC progression, serum levels of immunosuppressive (IL-10, IL-13, and IL-4) and pro-inflammatory (IL-6, TNF-α, and IL-1β) cytokines significantly increased as the disease progressed (Fig. 5A). FACS analysis revealed an influx of F4/80^+^CD11b^+^ inflammatory monocytes in peritoneal lavage from *Lect2*^−/−^ and *Lect2*^+/+^ mice. Notably, we discovered an enrichment of CD206^+^ cells in *Lect2*^−/−^ mice compared to *Lect2*^+/+^ mice (Fig. 5B). Moreover, a more robust accumulation of CD206^+^ cells in ascites was present in *Lect2*^−/−^ EOC compared with the *Lect2*^+/+^ EOC model (Fig. 5C), indicating the polarization of immunosuppressive M2-like TAMs infiltrating in *Lect2*^−/−^ EOC (*P* < 0.05; Fig. 5D). Furthermore, previous studies have highlighted MDSCs as critical drivers of immunosuppression in EOC, and increased MDSCs in EOC are associated with poor prognosis [17]. So, we characterized inflammatory monocytes and analyzed MDSC subsets in *Lect2*^−/−^ EOC. We found a more substantial accumulation in CD45b^+^/CD11b^+^ myeloid cells in *Lect2*^−/−^ EOC compared with *Lect2*^+/+^ EOC. Upon flow cytometry gating, we verified a higher abundance of Ly6C+/Ly6G− cells (M-MDSCs) (Fig. 5E). Notably, whereas the percentage of M-MDSCs in *Lect2*^−/−^ EOC was higher than in *Lect2*^+/+^ EOC (*P* < 0.01), the proportion of PMN-MDSCs (Ly6C^-^/Ly6G^+^) remained unchanged (*P* = 0.82; Fig. 5F).

### Lect2 altered gene expression of MDSC via the CD209a receptor and directly inhibited MDSC-mediated T-cell suppression

**Fig. 6:**
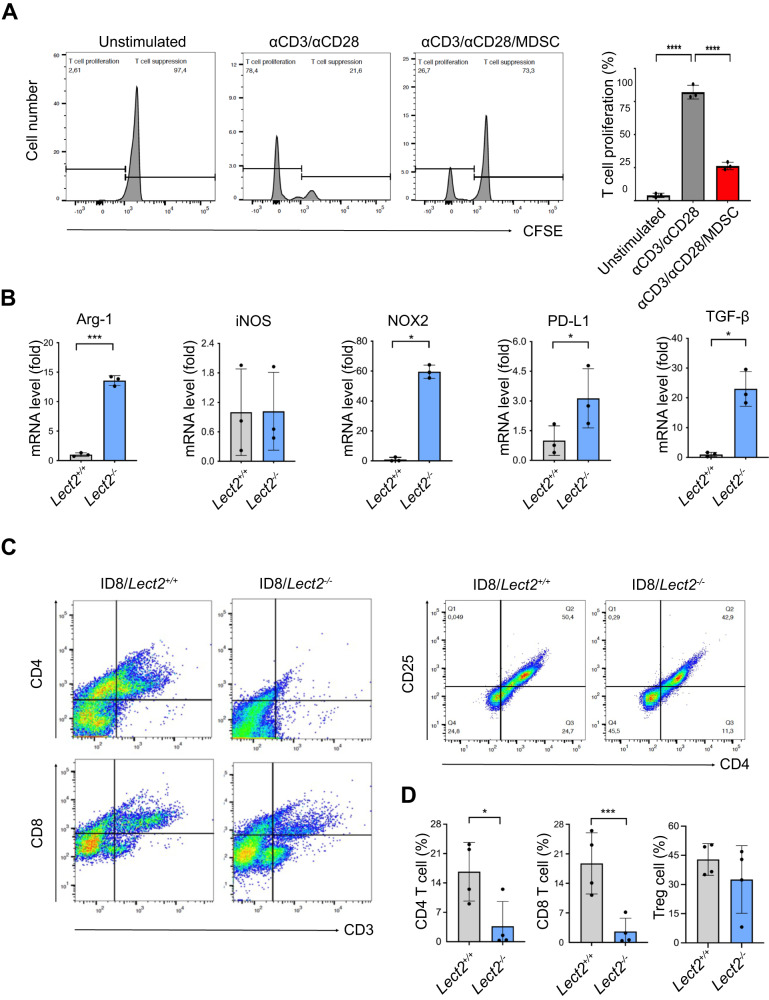
LECT2 inhibited MDSC-mediated T-cell suppression. **A** The representative histograms (left) from flow cytometry analysis show the CFSE intensity in unstimulated, anti-CD3/anti-CD28-stimulated, and stimulated/MDSC co-cultured CD8 T cells. Bar graphs (right) show the percentage of proliferating CD8 T-cell cells. MDSC was co-cultured with anti-CD3/anti-CD28-stimulated T-cell activation in the ratio of 2:1. An unstimulated T cell was used as a control. ****P < 0.0001. **B** The mRNA fold change of intracellular immune-suppressive mediators (Arg-1, iNOS, NOX-2, TGF-β, and PD-L1) in MDSCs extracted from the ascites of the ID8/*Lect2*^−/−^ or ID8/*Lect2*^+/+^ mice by qPCR. *P < 0.05 and ***P < 0.001; **C** The representative density plots from flow cytometry analysis show CD4, CD8, and Treg cell proportions in the ascites of ID8/*Lect2*^−/−^ and ID8/*Lect2*^+/+^ mice. **D** Bar graphs show CD4, CD8, and Treg cell percentages in the ascites of ID8/*Lect2*^−/−^ and ID8/*Lect2*^+/+^ mice (*n* = 4 per group). *P < 0.05 and ***P < 0.001. ***P < 0.001 and ****P < 0.0001.

To test the immunosuppressive effect of these MDSCs, we demonstrated that including MDSCs significantly suppressed Anti-CD3/Anti-CD28 mediated T-cell activation (*P* < 0.001; Fig. 6A). We further revealed that Arg-1 (*P* < 0.001), NOX-2 (*P* < 0.05), TGF-β (*P* < 0.05), and PD-L1 (*P* < 0.05) were significantly higher from *Lect2*^−/−^ EOC than *Lect2*^+/+^ EOC (Fig. 6B), supporting their potential for inhibiting the tumor-infiltrating lymphocytes and silencing the immune response. In line with these findings, the accumulation of ascites CD4^+^ T cells (*P* < 0.05) and CD8^+^ T cells (*P* < 0.001) were significantly reduced in *Lect2*^−/−^ EOC compared with *Lect2*^+/+^ EOC in both number and proportion, while the level of Treg (*P* = 0.33) remained unchanged (Fig. 6C, D).

**Fig. 7:**
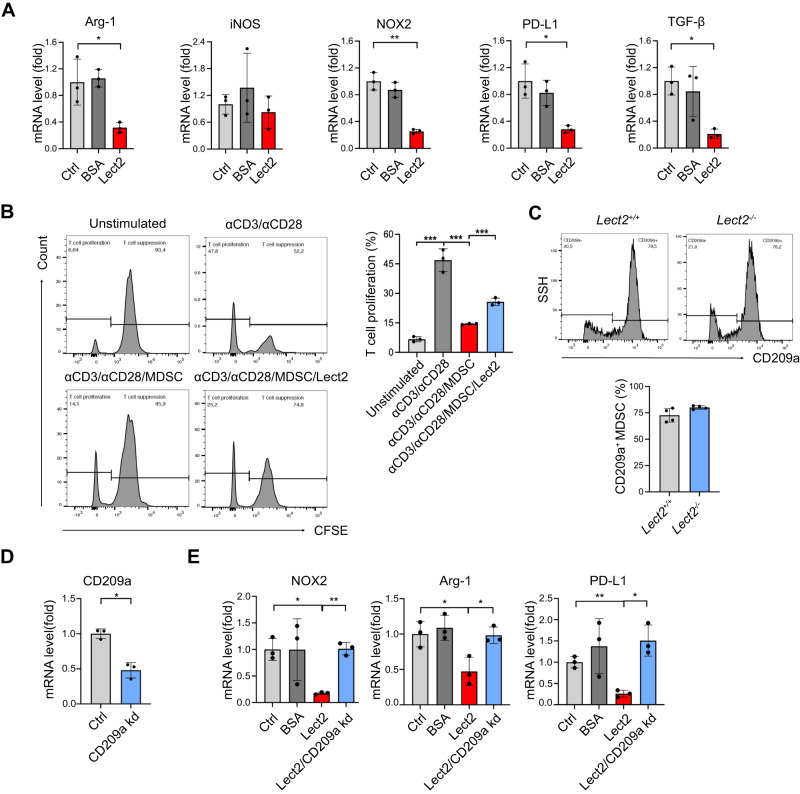
Lect2 altered gene expression of MDSC via the CD209a receptor. **A** The mRNA fold change by qPCR of the immunosuppressive profile in MDSCs after 96 h of in vitro stimulation with Lect2 (160 ng/ml). *P < 0.05 and **P < 0.01. **B** The representative histograms (left) from flow cytometry analysis show the CFSE intensity of CD8 T cells in different treatment groups. Bar graphs (right) show CD8 T-cell proliferation. An anti-CD3/anti-CD28-stimulated T cell without MDSC co-culture was used as a control. The stimulated T cells were co-cultured with MDSC (1:2) in the presence of Lect2 (80 ng/ml) or not. **C** The histogram from flow cytometry analysis and bar graphs showing CD209a expression in splenic MDSCs of *Lect2*^−/−^ or *Lect2*^+/+^ mice. **D** Q-PCR validation of the CD209a knockdown (CD209a kd) of the MDSCs in vitro. **E** The mRNA fold change of intracellular immune-suppressive mediators (NOX-2, Arg-1, and PD-L1) in MDSCs treated with control, BSA (80 ng/ml), Lect2 (80 ng/ml), and Lect2 in CD209a knockdown groups (each group *n* = 3).

We next determined whether Lect2 would modify immunoregulatory programs of MDSCs in vitro. Figure 7A demonstrates that the immunosuppressive profiles of MDSCs, including Arg-1 (*P* < 0.05), NOX-2 (*P* < 0.01), TGF-β (*P* < 0.05), and PD-L1 (*P* < 0.05), were significantly reduced by Lect2 supplement in the culture medium. Co-treatment of Lect2 significantly rescued MDSC-inhibited T-cell activation (Fig. 7B). Furthermore, Lect2 mainly interacts with two receptors, CD209a and c-Met. We identified a substantial expression of CD209a in MDSCs (Fig. 7C), whereas c-Met was not typically expressed in these cells (data not shown). To further demonstrate whether Lect2/CD209a axis mediated Lect2-induced MDSC immune modulation, CD209a expression was knocked down by transfection of MDSC with CD209a-specific siRNA (Fig. 7D). The lack of CD209a significantly prohibited Lect2-mediated NOX-2, Arg-1, and PD-L1 expression in MDSCs (Fig. 7E).

### Loss of Lect2 hindered the anti-cancer activity of immune checkpoint inhibitors

**Fig. 8:**
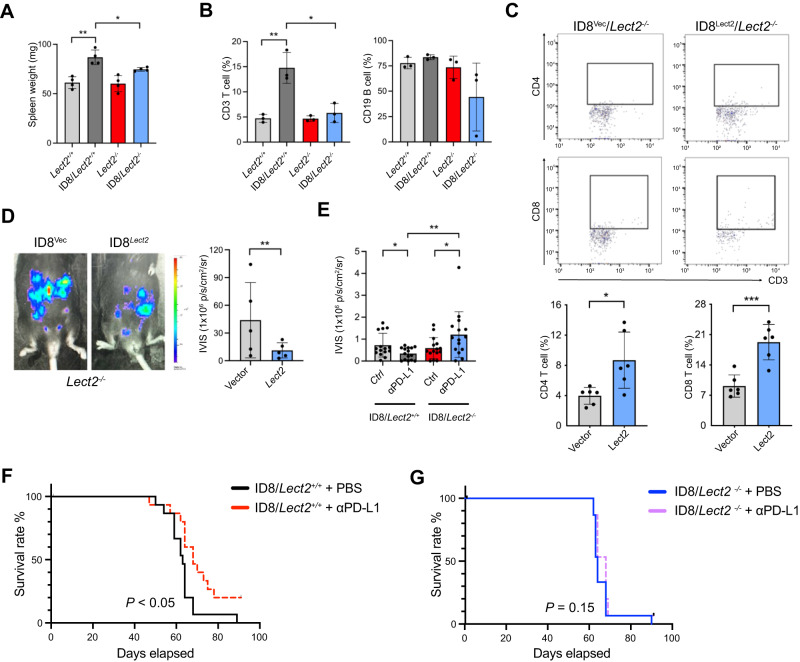
Reconstitution of Lect2 expression restores anti-tumor immunity. **A** The spleen weight of mice. The spleen weight is measured on day 35 after the ID8 injection. *P < 0.05 and **P < 0.01. **B** The splenic T-cell and B cell percentages of the mice as described in (**A**). **C** The CD4 and CD8 T cells in malignant ascites of the *Lect2*^−/−^ mice on day 56 after inoculation of ID8/vector control (ID8/Vec) or ID8/*Lect2* overexpression cells (ID8/*Lect2*). *P < 0.05 and ***P < 0.001. **D** The tumor burden of the *Lect2*^−/−^ mice was detected by the luciferase activity on day 56 in the ID8/*Lect2* and the ID8/Vec group. **P < 0.01. **E** The luciferase activity of the ID8/*Lect2*^+/+^ mice and ID8/*Lect2*^−/−^ mice treated with the anti-PD-L1 antibody on day 21 (*n* = 15 per group). *P < 0.05 and **P < 0.01. **F**, **G** Kaplan-Meier survival curves for *Lect2*^+/+^ and *Lect2*^−/−^ mice since day 0 of inoculation with 5 × 10^6^ ID8 cells, administered with anti-PD-L1 antibody since day 7 (*n* = 15 per group). *P < 0.05 and n.s. non-significant.

We examined splenic immune response in EOC tumor-bearing mice. While there was a significant increase in spleen weight in *Lect2*^+/+^ EOC compared to non-tumor-bearing *Lect2*^+/+^ mice (*P* < 0.01), no significant differences were observed between *Lect2*^−/−^ EOC and non-tumor-bearing *Lect2*^−/−^ mice. Apparent discrepancies occurred with spleen weight when comparing *Lect2*^−/−^ EOC to *Lect2*^+/+^ EOC (*P* < 0.05; Fig. 8A). Specifically, there was a substantial increase of T cells in the spleen from *Lect2*^+/+^ EOC compared to their non-tumor-bearing counterparts (*P* < 0.01; Fig. 8B), while splenic T-cell recruitment in *Lect2*^−/−^ EOC was negligible. In comparison, B cell recruitment in the spleen was not remarkable in tumor-bearing *Lect2*^+/+^ and *Lect2*^−/−^ mice (Fig. 8B). A lentiviral vector encoding the secreted mouse Lect2 protein was introduced into the ID8 cells in this context. Overexpression of *Lect2* resulted in a significant increase of ascitic CD4^+^ (*P* < 0.05) and CD8^+^ (*P* < 0.001) T cells in *Lect2*^−/−^ EOC (Fig. 8C). In turn, the ID8 tumor burden of *Lect2*^−/−^ mice was significantly reduced upon *Lect2* overexpression compared to the vector control (*P* < 0.01; Fig. 8D). Previous studies have reported that PD-1 or PD-L1-blockade therapy causes regression of ID8 EOC tumors [18]. We next tested whether the reduction of Lect2-mediated immune decline in the tumor microenvironment could impair PD-1/PD-L1 immunotherapy for EOC tumors. Two weeks after the initiation of therapy, treatment of α-PD-L1 antibody resulted in significant tumor inhibition in *Lect2*^+/+^ EOC (*P* < 0.05) in contrast to the tumor development in *Lect2*^−/−^ EOC (*P* < 0.05), demonstrating a significant difference in early tumor response to PD-L1blockade between *Lect2*^+/+^ EOC and *Lect2*^−/−^ EOC (*P* < 0.01; Fig. 8E). Blockade of PD-L1 resulted in tumor rejection in 20% (3 of 15) of the *Lect2*^+/+^ EOC mice, leading to significantly longer survival (*P* < 0.05; Fig. 8F). In contrast, α-PD-L1 antibody did not elicit rejection of *Lect2*^−/−^ EOC; tumors and ascites rapidly accumulated at approximately 28 days, leading to death at 63 to 70 days (*P* = 0.13; Fig. 8G).

## Discussion

Accumulating evidence supports a tumor-suppressive role of LECT2, given that uncontrolled inflammation is involved in cancer progression. LECT2 is mainly synthesized in the liver and secreted into the blood [8]. It should come as no surprise that LECT2 initially became a protein of interest in the carcinogenesis of HCC. Nevertheless, the precise functions and mechanisms of LECT2 in non-hepatic malignancies have been left unexplored and remain to be determined. In our cohort of EOC patients and a syngeneic murine EOC model, we demonstrated an increase in down-regulated LECT2 expression as the disease progressed. Previous studies have suggested that EOC cells can secrete various substances, including lipids, cytokines, hormones, and exosomes, to create pre-metastatic niches and modulate immunosurveillance [19, 20]. Future studies are warranted to explore the underlying mechanisms in this regard. Here, we showed that LECT2 inhibits EOC cell invasiveness by targeting HGF/c-Met signaling. More importantly, we revealed that LECT2 controls EOC progression, targeting the immune microenvironment. Using the murine ID8 EOC model, we verified that Lect2 directly acts on immature myeloid cells and that loss of Lect2 fosters MDSCs, together with M2-polarized macrophages, impairs innate and adaptive anti-tumor immunity. Indeed, we provided evidence that loss of Lect2 substantially impairs the therapeutic efficacy of checkpoint blockade in cancer immunotherapy for murine ID8 EOC, which suggests that loss of Lect2 poses a significant obstacle to EOC immune therapy.

We have previously identified that LECT2 directly binds to the α-chain of the c-Met receptor and induces a phosphatase-dependent inhibition of HGF/c-Met signaling in HCC [12]. C-Met is overexpressed in lung, breast, ovary, colon, and pancreas cancers, among others [21], suggesting the LECT2/c-Met regulatory axis may be a common pathway in human cancers. Indeed, LECT2 was found to suppress tumor metastasis by targeting the HGF/c-Met signaling in pancreatic ductal adenocarcinoma (PDAC) [22]. Also, we have recently characterized a suppressive role of LECT2 in non-small cell lung cancer (NSCLC) progression via antagonizing c-Met and epidermal growth factor receptor (EGFR) [23]. In line with these findings, we observed a significant impact of LECT2 on the HGF/c-MET signaling in EOC cells and the abrogation in cell adhesive, migratory, invasive, and metastatic potentials of EOC cells. Given that c-Met targeting is essential in inhibiting the aggressive properties of EOC where HGF signaling is constitutively activated, LECT2 may have the potential to serve as an alternative strategy in developing c-Met antagonists in addition to the current small molecule inhibitors.

Angiogenesis is essential for tumor expansion and transcoelomic metastasis of EOC. Vascular endothelial growth factor (VEGF) selectively accumulates in ascites and occurs in advanced stages of EOC, holding a pivotal role in the angiogenic process of EOC, mainly by regulating neovascularization and vascular permeability. In EOC, the anti-angiogenic potential of LECT2 has been addressed. HGF stimulates endothelial cells directly through the c-MET receptor and indirectly by facilitating the expression of other angiogenic factors represented by VEGF [24]. VEGFR2 and Tie1 have been proposed as membrane receptors of LECT2 in endothelial cells [25, 26]. Besides, the formation of malignant ascites is partly the result of an immunogenically induced host response to intraperitoneal metastases. We found that the loss of Lect2 enriches ascitic CD206^+^ TAMs in the murine EOC model. CD206^+^ TAMs are intensely involved in EOC tumor angiogenesis and ascites formation in mice [27], in line with our current data showing that CD206^+^ TAMs accelerated the growth of ID8 peritoneal tumors and ascites formation in *Lect2*^*−/−*^ mice. Although further investigations are required, our finding of a significant decrease in ascites volume in tumor-bearing mice with high Lect2 levels suggests that maintaining the amount of regional or circulating Lect2 may help ease the ascites formation by acting on these receptors.

Our data demonstrate that Lect2 disrupts the homeostasis of the immune environment in the ID8 EOC model. Macrophages are a significant component of the leukocyte infiltrates present in EOC and orchestrate cancer-related inflammation [28]. Consistent with previous reports that HGF-cMet signaling shifts M1 macrophages toward an M2-like phenotype [29], our data show that loss of Lect2 contributes to M2-subtype TAM polarization, which might result in taming adaptive protective immunity and paving the way to metastasis [28]. Several clinical studies have reported that low M2 TAM density was associated with an increase in progression-free survival and overall survival in advanced EOC, and extended survival was observed for patients with a high ratio of M1 (HLA-DR^+^/iNOS^+^) to M2 (CD163^+^/VEGF^+^) TAMs in EOC [30]. TAMs have been shown to hamper T-cell responses in EOC by expressing immune checkpoints like B7-H4 and PD-1, exacerbating an immunosuppressive environment [31, 32].

Tissue-resident macrophages have distinct functions in promoting transcoelomic metastasis of EOC [33]. However, it has long been held that monocyte-derived macrophages serve as a reservoir for macrophage replenishment and are recruited during tumorigenesis [23, 34]. Our data demonstrated that M-MDSC enriches the ascites microenvironment of *Lect2*^−/−^ EOC, suggesting they could function as the Tumor associated macrophages (TAM) precursor. Moreover, the salient features of MDSC are their ability to inhibit T-cell function and secrete various pro-inflammatory mediators and growth-stimulating cytokines, indicating that MDSC is the critical driver of immunosuppression in EOC [35]. Like many other types of human cancers, high levels of MDSC in human EOC, specifically M-MDSC, have also been associated with poor prognosis [17, 36]. Different pathways are involved in recruiting monocytes/macrophages into distinct tumor microenvironments. In EOC, for example, both CXCL12/CXCR4 and VEGF/VEGFR2 pathways have been shown to attract MDSC into the ascites microenvironment [36, 37]. Thus, it is conceivable that LECT2 may suppress MDSC accumulation triggered by the VEGF signaling in EOC. An alternative possibility is that LECT2 may interfere with MDSC expansion through c-Met signaling, with supportive evidence from previous studies demonstrating that the HGF/c-Met pathway triggers MDSC expansion from multipotent human mesenchymal stromal cells [38].

The generation of MDSC requires the pathological activation of immune regulatory programs to inhibit adaptive immunity. During unresolved inflammation in cancer, the most prominent biochemical features implicated in MDSC suppressive activity include increased expression of Arg1, iNOS, NOX-2, and primarily anti-inflammatory cytokines [39–43]. Our data showed that pathological activation of MDSC, when exposed to the EOC-conditioned media, resulted in the expression of Arg1, iNOS, NOX-2, and TGF-β. We found that the presence of Lect2 significantly inhibits Arg1, NOX-2, and TGF-β expression in MDSC, suggesting Lect2 directly suppresses pathological activation of MDSC, through membrane CD209a signaling. In addition to IL-10 and VEGF in the EOC tumor microenvironment [44], our results indicate that Lect2 is another novel modulator of PD-L1 expression in MDSC. Overall, these alterations may reduce reactive oxygen species (ROS) and prevent l-Arg depletion, leading to an increase in the expression of the T-cell receptor and restoration of T-cell responses [45, 46].

In EOC, myeloid cells are one of the critical determinants of immune suppression. Loss of Lect2 fosters the accumulation/activation of M-MDSC, leading to the presence of M2-like TAMs and pro-tumorigenic cytokines (IL-4, IL-6, IL-10, and TGF-β) that can impair the activity of T cells in the fight against cancer. PD-1 is one of the most potent examples of T-cell immune checkpoint molecules and has been successfully targeted to treat various recalcitrant cancers. The PD-1 pathway is highly relevant and is active early in establishing the murine ID8 EOC model. Blockade of the PD-1/PD-L1 pathway prevents immune decline in the EOC microenvironment and causes tumor regression [18]. However, our data demonstrated that the efficacy of PD-1/PD-L1 blockade in preventing immune decay and tumor rejection significantly diminished in *Lect2*^*−/−*^ mice, suggesting multiple immunological brakes need to be lifted to augment the effective immune system response against EOC. These observations indicate the pivotal role of Lect2 in the interplay between innate and adaptive immunity for successful immunotherapy in EOC.

In summary, we have elucidated an emerging role of LECT2 in EOC, which suppresses tumor progression. LECT2 can bind directly to several receptors and mediate distinct molecular pathways in various cell settings. Decreased expression of LECT2 during EOC progression has profound effects on both tumor cells and tumor microenvironment and potentially impedes the success of immunotherapy in EOC. While unknown mechanisms and interactions still necessitate further research, our findings offer insights into developing a novel therapeutic strategy for EOC. In this context, reintroducing LECT2 is a rational approach to efficiently circumvent the immunosuppressive EOC tumor microenvironment and thus boost the likelihood of response to immune checkpoint blockade.

## Materials and methods

### Cell culture

The human ovarian cancer cell line SKOV-3 and human liver cell line SK-Hep1 were obtained from the American Type Culture Collection. The mouse epithelial ovarian cancer cell line ID8 was obtained from Dr. Kathy Roby (The University of Kansas Medical Center). SKOV-3 cells were grown in McCoy’s 5A (Sigma M4892) medium containing 10% fetal bovine serum (FBS) (Life Technologies). ID8 cells were cultured in DMEM medium supplemented with 10% FBS and 1×insulin/transferrin/selenium (ITS) (Sigma I1884). SK-Hep1 cells were grown in DMEM medium containing 10% fetal bovine serum (Life Technologies). All cells were grown in a humidified incubator with 5% CO_2_ at 37 °C. All cells were routinely authenticated based on morphologic and growth characteristics and by a short tandem repeat (STR) analysis and confirmed to be free of mycoplasma.

### RNA isolation and reverse transcription polymerase chain reaction

According to the manufacturer’s instructions, total RNA from cells was isolated using TRIzol reagent (Invitrogen), and cDNA was synthesized from 1 μg TRIzol-extracted RNA from each sample using the iScriptTM cDNA synthesis kit (BioRed); quantitative reverse transcription-PCR was carried out using the KAPA SYBR® FAST One-Step qRT-PCR Master Mix (2X) Kit (KK4650) and Bio-Rad iQ5 detection system. Forward and reverse primers are shown in Supplementary Table 1. Gene expression values were calculated relative to GAPDH expression for each sample.

### Western blot and immunohistochemical staining analysis

Cells were lysed in NETN lysis buffer containing a protease inhibitor cocktail (Sigma). Equal amounts of proteins were separated by SDS-PAGE and transferred to a polyvinylidene fluoride membrane. After blocking, Western blot analysis was carried out using the following primary antibodies: Met (#4560, Cell Signaling), phospho-Met (1234/1235) (#3077, Cell Signaling), Akt1 (sc-8312, Santa Cruz), p-Akt1 (sc-33437, Santa Cruz), Erk1 (sc-93, Santa Cruz), p-Erk1/2 (sc-16982, Santa Cruz), and α-tubulin (T5168, Sigma-Aldrich). IHC staining of phospho-Met was carried out with anti-p-Met (1234/1235) (#3077, Cell Signaling).

### *Lect2*-knockout (KO) mice

*Lect2*-KO mice were produced as described previously [9] and kindly provided by Dr. Yamagoe (National Institute of Infectious Diseases, Tokyo, Japan). Heterozygous mice (*Lect2*^+/−^) were intercrossed to generate mice with different genotypes in the National Laboratory Animal Center (Tainan, Taiwan). Littermates were genotyped and divided into groups.

### Allograft mouse model

Protocols for all animal studies were approved by the National Taiwan University College of Medicine Institutional Animal Care and Use Committee. Six to eight weeks old female *Lect2*^+/+^, *Lect2*^+/−^, and *Lect2*^−/−^ mice (C57BL/6J background) were used and randomly grouped for peritoneal dissemination assays. 5 × 10^6^ ID8/luciferase (ID8/Luc) cells were suspended in PBS and intraperitoneally injected into the mice. The sample size of 8 in each group was calculated to have an 80% probability of showing a statistically significant difference (*P* < 0.05). There are no bindings to the investigators. The tumor burden was monitored and quantified using a non-invasive bioluminescence system (IVIS-Spectrum). Seven to ten weeks after the injection, the mice were sacrificed, and the ascites of each mouse were collected and measured. The number of tumor nodules in the abdominal cavity was counted in multiple experiments. The calculations encompass all nodules within the peritoneal cavity, spanning the diaphragm, mesentery, and peritoneum. A portion of nodules from each was resected for Western blotting analysis. For survival analysis, 200 μg anti-mouse PD-L1 (programmed death-ligand 1) (BioCell BE0101) was injected four times/week for four weeks into the ID8/Luc-bearing mice. The mice were monitored to establish a 13-week survival curve, and the surviving mice were sacrificed after completing the experiments.

### T-cell suppression assay

A single-cell suspension was prepared from the spleens of C57BL/6 mice, and CD8α^+^ T cells were isolated using the EasySep mouse CD8α^+^ T-Cell Isolation Kit (Stem Cell Technology). Sorted T cells were labeled with carboxyfluorescein diacetate succinimidyl ester (CFSE, Invitrogen) and plated at 1 × 10^5^ cells /well in 96-well plates. And then, the cells were stimulated with 2 µg/ml CD3ε (eBioscience), 1 µg/ml CD28 (eBioscience), 1 µl/ml anti-CD3/anti-CD28 beads (Invitrogen). Enriched Ly6G^+^ or Ly6C^+^ myeloid-derived suppressor cells (MDSCs) were obtained using the Myeloid-Derived Suppressor Cell Isolation Kit (Miltenyi Biotec) from spleens of C57BL/6 mice inoculated with ID8 cells for 6 weeks. Sorted MDSCs were added to the stimulated T cells at a 2:1 ratio of MDSCs (2 × 10^5^) to T cells (1 × 10^5^). After 72 h, dilution of CFSE was detected by flow cytometry as a measure of T-cell proliferation. To that end, T cells were additionally stained with anti-CD3-APC (Biolegend). Data was collected using the LSRII flow cytometer (BD Biosciences) and analyzed with FlowJo V10.6 (Tree Star Inc.). For the Lect2 functional test, we added recombinant mouse Lect2 protein (Origene) to the well (80 ng/ml).

### Cell surface and intracellular staining by flow cytometry

Cells were subjected to fluorescence-activated cell sorting (FACS) analysis with specific antibodies. The antibodies used for cell surface staining were: anti-mouse CD45 and anti-mouse CD206 (TONBO); anti-mouse CD11b, anti-mouse CD11c, anti-mouse CD8, anti-mouse F4/80, and anti-mouse CD209a (eBioscience); anti-mouse CD4, anti-mouse CD3, anti-mouse Ly6C, and anti-mouse Ly6G (Biolegend). Data were acquired with an LSRII flow cytometer (BD Biosciences) and analyzed with FlowJo V10.6 (Tree Star Inc.).

### Mouse Lect2 treated MDSCs

5 × 10^5^ ID8 cells were seeded in a 10 cm dish and cultured with RPMI 1640 medium for 48 h to generate an ID8-conditioned medium. The MDSCs of the spleen of *Lect2*^+/+^ female mice were cultured for 48 h in RPMI 1640 medium with 10% FBS, 1% penicillin, and streptomycin, and to the medium was added BSA or Lect2 recombinant protein in 24-well plates. After 48 h, the medium was replaced with ID8-conditioned medium and replenished with BSA or Lect2 recombinant protein for another 48 h; the MDSCs were then harvested to extract the mRNA to run qPCR.

### Ascites analysis

A written informed consent for participation was obtained from all patients and healthy volunteers. The study was authorized by the local ethics committees of the National Taiwan University Hospital (Taipei, Taiwan). Ascites aspirated were collected and sent for the concentration of LECT2.

### Statistical analysis

The data were presented as the mean ± standard deviation (SD). The Student’s *t*-test was used to compare data between the two groups. Statistical analyses of clinicopathological data were performed by the chi-square exact test. *P* values of less than 0.05 were statistically significant.

### Supplementary information

Supplemental materal

## Data Availability

The data that support the findings of this study are available from the corresponding author, LHW, upon reasonable request.
