# Supplementary material for: Loss of LECT2 promotes ovarian cancer progression by inducing cancer invasiveness and facilitating an immunosuppressive environment
Source: Oncogene. 2024 Jan 4;43(7):511–23. doi: 10.1038/s41388-023-02918-w (PMC10857938; doi:10.1038/s41388-023-02918-w)
Supplement: Supplementary file 1 — Supplemental materal [file 41388_2023_2918_MOESM1_ESM.docx]

**Supplement**

**Material and Methods**

***In vivo* and *in vitro* adhesion assays**

ID8 cells were fluorescently labeled with 10 μM CMFDA (Molecular Probes). After recovery for 30 minutes, 5×10^6^ cells were suspended as single cells in a volume of 100 μl PBS and intraperitoneally injected into *Lect2*^+/+^ or *Lect2*^-/-^ mice. After 24 hours, mice were sacrificed, and adherent cells on the peritoneum, mesentery, and diaphragm were compared between different genotype mice. For the in vitro adhesion assay, SKOV-3 or ID8 cells were fluorescently labeled with 10 μM CMFDA. After recovery for 30 minutes, 3×10^4^ cells/well were plated in a 24-well plate precoated with collagen type 1 (50 μg/ml), fibronectin (15 μg/ml), or Matrigel (20 μg/ml). After incubation for 30 minutes at 37°C, cells were washed and fixed. The adherent cells were counted under a microscope (Leica Microsystems).

**Migration and Invasion Assay**

Migration assays were performed using transwell inserts for a 24-well plate containing 8 μm pores (Millipore). Matrigel (BD Labware) coated filters were used for the invasion assay. Cells (2×10^4^) were plated into 200 μl of serum-free medium with 1 % FBS in the upper chamber, and the lower chamber was filled with 800 μl of serum-free medium with 10 % FBS. After 24 hours in culture, cells were fixed in methanol for 15 minutes and then stained with 0.05% crystal violet in PBS for 15 minutes. Cells on the upper side of the filters were removed with cotton-tipped swabs, and the filters were washed in PBS. Cells on the underside of the filters were viewed and counted by microscopic inspection. Each clone was plated in triplicate per experiment, and each experiment was repeated at least three times.

**Table**

Supplement Table 1

| **Primer** | **Sequence** |
| --- | --- |
| β-actin forward | AGAGGGAAATCGTGCGTGAC |
| β-actin reverse | CAATAGTGATGACCTGGCCGT |
| Arg-1 forward | CAAGACAGGGCTCCTTTTCAG |
| Arg-1 reverse | GTAGTCAGTCCCTGGCTTATGG |
| iNOS forward | CAGCTGGGCTGTACAAACCTT |
| iNOS reverse | CATTGGAAGTGAAGCGTTTCG |
| NOX2 forward | AACTGTATGCTGATCCTGCTGC |
| NOX2 reverse | GTTCTCATTGTCACCGATGTCAG |
| TGF-β forward | CCTCCCCCATGCCGCCCTCG |
| TGF-β reverse | CCAGGAATTGTTGCTATATTTCTG |
| PD-L1 forward | ATTGCTCCTTGACTGCTGGCTG |
| PD-L1 reverse | TTCTGGGTTTCCTCCTCCTTTCC |

Supplement Table 2. Demographic data of clinical epithelial ovarian cancer patients (n=84)

| Age | 55.68 ± 11 |
| --- | --- |
| Cell type |  |
| high-grade serous | 57 |
| clear cell | 22 |
| endometrioid | 8 |
| FIGO stage |  |
| I | 18 |
| II | 3 |
| III | 49 |
| IV | 14 |
| Malignant ascites | 19 |

**Supplement Figure 1**

The suppression ability of recombinant LECT2/Lect2 proteins in the adhesion assays of HGF-induced SKOV-3 and ID8 cells. HGF (30 ng/ml), recombinant LECT2/Lect2 (200 ng/ml), and Fc (200 ng/ml) were used as indicated. **P* < 0.05, ***P* < 0.01, and ****P* < 0.001
